# Metagenomics-metabolomics analysis of microbial function and metabolism in petroleum-contaminated soil

**DOI:** 10.1007/s42770-023-01000-7

**Published:** 2023-05-10

**Authors:** Yong-Quan Li, Ying Xin, Caili Li, Jin Liu, Tao Huang

**Affiliations:** 1 School of Medicine, Northwest Minzu University, Lanzhou, China; 2 Key Laboratory of Environmental Ecology and Population Health in Northwest Minority Areas, State Ethnic Affairs Commission, Lanzhou, China

**Keywords:** Petroleum, Soil, Pollution, Metabolism, Microorganism

## Abstract

Contamination of soil by petroleum is becoming increasingly serious in the world today. However, the research on gene functional characteristics, metabolites and distribution of microbial genomes in oil-contaminated soil is limited. Considering that, metagenomic and metabonomic were used to detect microbes and metabolites in oil-contaminated soil, and the changes of functional pathways were analyzed. We found that oil pollution significantly changed the composition of soil microorganisms and metabolites, and promoted the relative abundance of *Pseudoxanthomonas*, *Pseudomonas*, *Mycobacterium*, *Immundisolibacter,* etc. The degradation of toluene, xylene, polycyclic aromatic hydrocarbon and fluorobenzoate increased in Xenobiotics biodegradation and metabolism. Key monooxygenases and dioxygenase systems were regulated to promote ring opening and degradation of aromatic hydrocarbons. Metabolite contents of polycyclic aromatic hydrocarbons (PAHs) such as 9-fluoronone and gentisic acid increased significantly. The soil microbiome degraded petroleum pollutants into small molecular substances and promoted the bioremediation of petroleum-contaminated soil. Besides, we discovered the complete degradation pathway of petroleum-contaminated soil microorganisms to generate gentisic acid from the hydroxylation of naphthalene in PAHs by salicylic acid. This study offers important insights into bioremediation of oil-contaminated soil from the aspect of molecular regulation mechanism and provides a theoretical basis for the screening of new oil degrading bacteria.

**Supplementary Information:**

The online version contains supplementary material available at 10.1007/s42770-023-01000-7.

## Introduction

Oil is the most important source of energy and chemical raw material in today’s society [1]. However, oil leakage might happen in any stage of the oil industry from exploitation, transportation, to storage and processing, causing serious environmental pollution and ecological disasters [2, 3]. Oil destroys the structure and affects the permeability of soil [4, 5]. In the process of natural degradation of petroleum, toxic intermediate products are formed, which are poisonous to plant roots [6]. In addition, a large amount of oxygen is consumed during the process, which inhibits plant roots from respiration, contributing to rot disease and even death. As a complex compound, petroleum can react with nitrogen, phosphorus and other chemicals, depleting these elements in the soil and resulting in crop losses [7]. Polycyclic aromatic hydrocarbons (PAHs) and other components that are difficult to degrade remain in the body of producers, the first level in a food chain. With the gradual expansion of food chain and food web, PAHs are transferred and accumulated in the upper level, causing damage to human health [2]. Furthermore, with precipitation and runoff, polluted areas would expand to a larger scale from soil to even water bodies. Leaked oil would also cause permanent toxicity as it leads to genetic mutation of organisms [8, 9].

The abundance and composition of soil microorganisms are an important aspect of soil health [10–12]. The existence and concentration of pollutants will change the population, community structure and ecological function of soil microorganisms [13]. The structure of soil microbial community has a strong correlation with soil parent material, environmental factors and crop planting conditions [14, 15]. The change of flora is closely related to the expression of function. When oil spills into ecological environment, it affects the structure and metabolism of the microbial community [16]. Zhang et al. [17] found that the proportion of carbon and nitrogen in soil and bioavailable organic carbon (BAC) are one of the important factors regulating microbial metabolism, and their consumption is positively correlated with oil removal rate. Adjusting soil pH, aeration and water content can maximize the utilization rate of BAC, promote the degradation of oil and maintain the stability of soil microbial abundance and community composition. At the same time, it was found that the accumulation of fatty acids affected the microbial activity and limited the oil degradation efficiency to a certain extent.

Previous studies indicate that microbial and metabolic composition of soil play an irreplaceable role in the petroleum degradation system. The study of microorganisms in soil environment can help to understand the complex composition and diverse functional characteristics of the environment [18]. At present, metagenomic and metabonomic analyses have become powerful tools to study microbial flora function and metabolite changes. Bao et al. [19] analyzed the microbial community of oil-contaminated soil by metagenomic sequencing and found a high diversity of microbial community and metabolites. Functional studies showed that the expression of enzymes increased significantly during the degradation of a series of exogenous aromatic compounds, indicating that soil microbial community has great potential in the degradation of heterologous aromatic compounds.

Despite all the above, research on gene functional characteristics, metabolites and distribution of microbial genomes in oil-contaminated soil is limited, and the regulatory mechanism is not clear. Therefore, it is of great significance to study the changes of microbial community structure, functional genes and metabolic pathways in the process of natural degradation of petroleum. Furthermore, this study intends to screen the degradation strains of organic pollutants, find characteristic target functional genes of different pollution degrees and carry out bioremediation of oil-polluted soil. There are a large number of antibiotics in the soil, producing bacteria and making the soil a resistance pool [20]. This study seeks to explore resistance genes in the soil microbial community and provide a reference for further research. Based on this, metagenomics sequencing technology was used to study the effects of oil pollution. Metabolomics is considered a research method that performs qualitative and quantitative analyses of all metabolites in specific biological samples under limited conditions [21]. We therefore adopted metabolomics to explore changes in the composition of soil metabolites, as well as accumulation pathways of the metabolites.

## Materials and methods

### Sample collection

The soil samples collected in the experiment were from the loess high crude oil wellhead and nearby areas in Qingcheng County, Huachi County and Xifeng District in Changqing Oilfield (Qingyang City, Gansu Province) [19, 22]. The oil-contaminated topsoil (0–20 cm) was collected as experimental soil, and the nearby unpolluted topsoil was collected as control soil (soil samples with less than 500 mg/kg of petroleum hydrocarbon content were regarded as clean soil). Soils were collected from multiple sites and then mixed. Plant roots, gravels and other impurities were removed. The soils were put into sterile tubes, transported to the laboratory with dry ice and then stored at − 80 °C. 

### Experimental design

A total of 20 soil samples were collected and divided into two groups: (I) 10 samples uncontaminated by oil (Group C); (II) 10 samples contaminated by oil (Group S). All samples were prepared for further analysis.

### Sequencing and bioinformatics analysis of metagenome

Soil genomic DNA was extracted with the MP Bio Fast DNA Spin Kit for soil (SDS/mechanical lysis). Paired-end sequencing (2 × 150 bp) was performed using Hiseq 6000platform (Illumina). Clean data were obtained after removing all reads containing adapters, N and low quality reads. SOAPdenovo software was used for assembly analysis, after which gene prediction and abundance analysis were performed based on the assembly results. Genes were mapped with KEGG database, and unigenes were matched with bacteria, fungi, archaea and viruses extracted from NR database (v.2018.01) of NCBI by Diamond software. Species annotation and abundance results were obtained, and top 10 species with the largest relative abundance were selected. R software (v.3.6.0) was used to count the number of species and visualization. The ade4, vegan and ggplot2 packages were used for principal component analysis (PCA) and non-metric multidimensional scaling (NMDS). The alpha diversity indices were calculated by using QIIME2 software (v. 2021.2) to evaluate the species richness and diversity differences of the microbial communities in each sample. The different species between different groups were detected by rank sum test, and species with significant differences between groups were screened by linear discriminant analysis (LDA). CARD database was used to classify resistance mechanism of resistance genes. Data between the two groups were analyzed by unpaired *t* test (R, v.3.6.0), and the differences were considered significant when *P* value < 0.05.

### Metabolite extraction and GC-TOF-MS analysis

To investigate differences in metabolic profiles among the samples, 1000 ± 5 mg of each of the 20 soil samples were transferred to a 5-mL tube with 1 mL of pre-cold extraction mixture (methanol/dH2O (v:v) = 3:1) and 1 mL of ethanoic acid. The sample was vortexed for 30 s and homogenized with ball mill for 4 min at 35 Hz. One milliliter of pre-cold extraction mixture (methanol/dH_2_O (v:v) = 3:1) was added after centrifugation. After evaporation in a vacuum concentrator, 60 μL of methoxyamination hydrochloride (20 mg/mL in pyridine) was added, derivatized by 80 μL of BSTFA regent (1% TMCS, v/v) at 70 °C. All samples were then analyzed by gas chromatograph coupled with a time-of-flight mass spectrometer (GC-TOF–MS). GC-TOF–MS analysis was performed using an Agilent 7890 gas chromatograph coupled with a time-of-flight mass spectrometer. A DB-5MS capillary column was used. One microliter aliquot of sample was injected in splitless mode. With helium as the carrier gas, the front inlet purge flow was 3 mL/min, and the gas flow rate through the column was 1 mL/min. The initial temperature was kept at 50 °C for 1 min, after which raised to 310 °C at a rate of 10 °C min^−1^, and then kept for 8 min at 310 °C. Temperatures for injection, transfer line and ion source were 280 °C, 280 °C and 250 °C, respectively. The energy was − 70 eV in electron impact mode. The mass spectrometry data were acquired in full-scan mode with the m/z range of 50–500 at a rate of 12.5 spectra per sec after a solvent delay of 6.35 min.

### Metabolomics data analysis

The final dataset containing the information of peak number, sample name and normalized peak area was imported to SIMCA16.0.2 software package (Sartorius Stedim Data Analytics AB, Umea, Sweden) for multivariate analysis. Data were scaled and logarithmic transformed to minimize the impact of both noise and high variance of the variables. The first principal component was analyzed by orthogonal projections to latent structures-discriminate analysis (OPLS-DA) modeling. To check the robustness and predictive ability of the OPLS-DA model, a 200 times permutations was further conducted. Afterward, the *R*^2^ and *Q*^2^ intercept values were obtained. Furthermore, the value of variable importance in the projection (VIP) of the first principal component in OPLS-DA analysis was obtained, which summarized the contribution of each variable to the model. The metabolites with VIP > 1 and *P* value < 0.05 (*t* test) were considered as significantly changed metabolites. In addition, databases including KEGG (http://www.genome.jp/kegg/) and MetaboAnalyst (http://www.metaboanalyst.ca/) were used for pathway enrichment analysis.

## Results

### Petroleum pollution had significantly changed the diversity and composition of microorganisms in soil

**Fig. 1:**
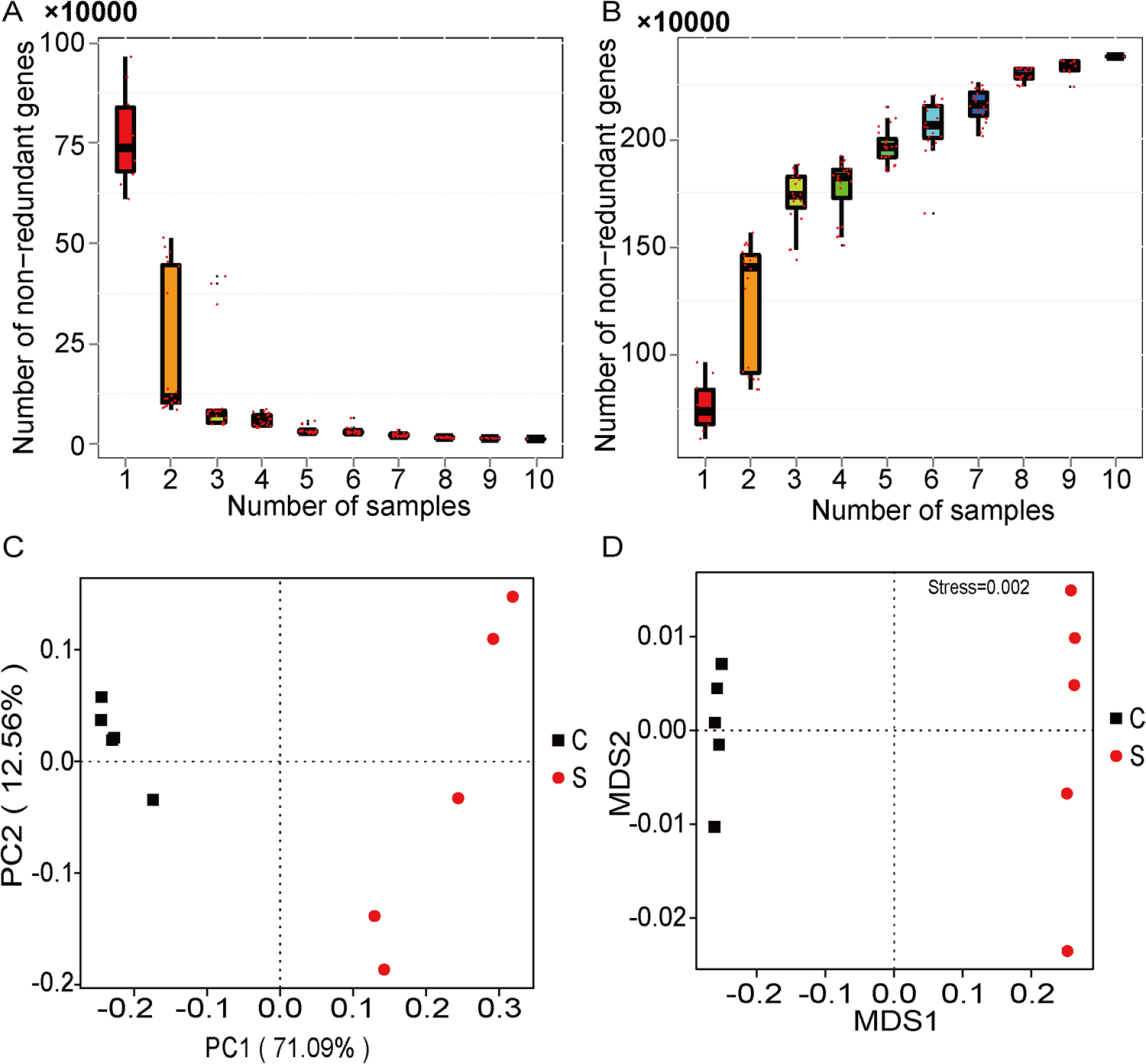
Oil pollution changes the composition of the whole microorganism in soil. **A, B** Represent the dilution curve of core and pan genes in contaminated and uncontaminated soil, respectively. **C** PCA based on Bray–Curtis. **D** NMDS analysis based on species abundance tables of different taxonomic levels. The degree of difference between different samples is reflected by the distance between points. The more similar the species composition of samples, the closer they are in the graph. S: oil pollution, C: control

The dilution curve of core and pan genes is close to flat (Fig. 1A, B). It shows that as the number of sequencing samples increases, the number of genes in the uncontaminated and contaminated soil gradually stabilizes, and the amount of sequencing samples is sufficient. In order to study the similarities of microbial composition between the two groups, PCA and NMDS cluster analysis were conducted based on Bray–Curtis distance matrix, and the cluster tree of samples was constructed. Significant differences were found in the overall composition of soil microorganisms between the uncontaminated and contaminated samples (Fig. 1C, D). Among the two algorithms, the PC1 distribution with the largest contribution rate revealed changes of 71.09%, and the PC2 distribution 12.56%. These results indicate that the composition of soil microorganisms was changed after contamination by oil.

**Fig. 2:**
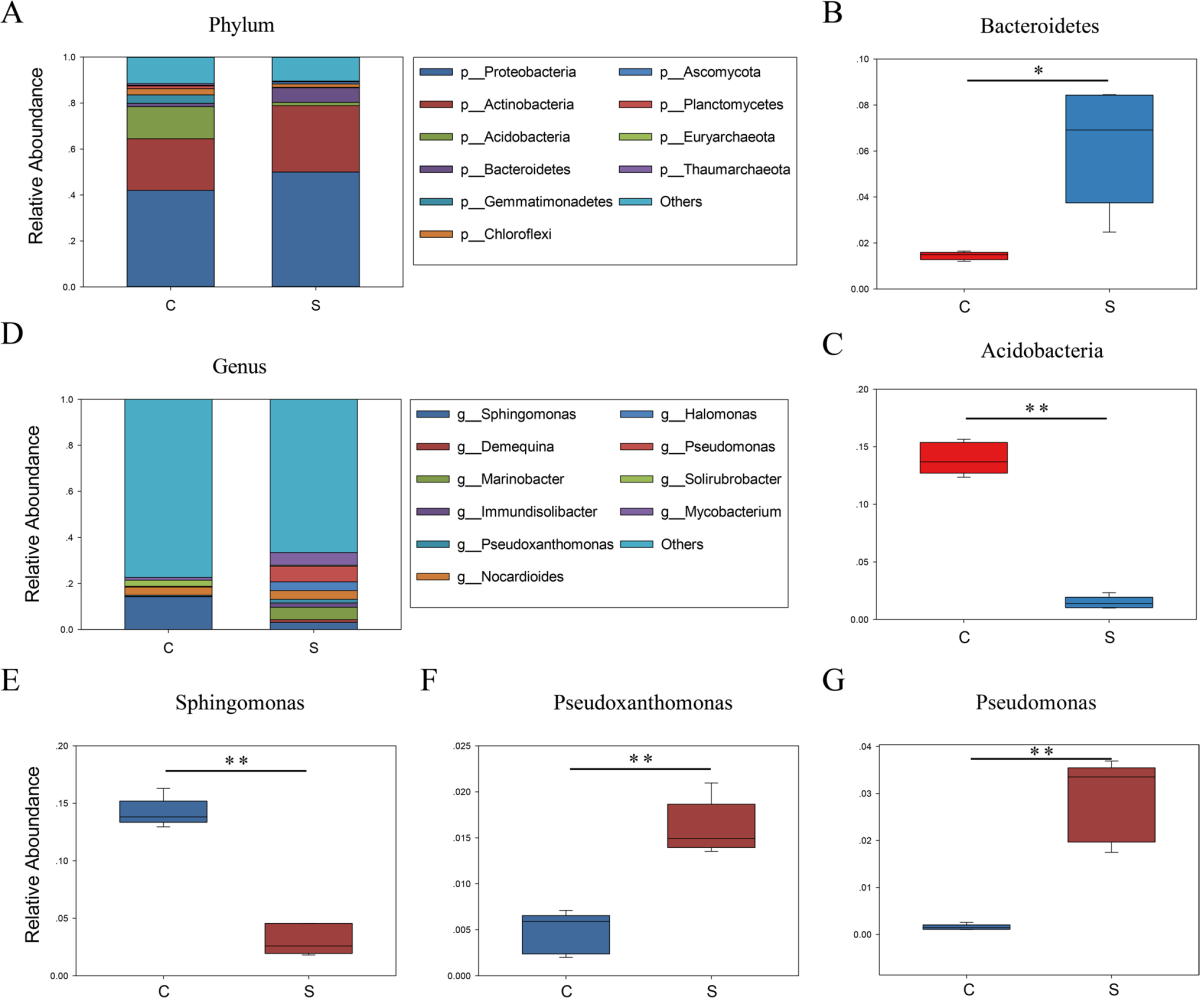
Composition of microorganisms in petroleum-contaminated soil at phylum and genus levels. **A** The relative contribution of the top 10 phyla in each group. **B, C** Represent the relative abundances of *Bacteroidetes* and *Acidobacteria,* respectively. Data were analyzed by *t* test (*P < 0.05; **P < 0.01). **D** The relative contribution of the top 10 genera in each group. **E–G** Represent the relative abundances of *Sphingomonas*, *Pseudoxanthomonas* and *Pseudomonas,* respectively. Data were analyzed by *t* test (*P < 0.05; **P < 0.01). S: oil pollution, C: control

In order to explore the specific distribution and composition of microorganisms in contaminated soil, we further analyzed the composition of flora at phylum and genus level. In uncontaminated soil, *Proteobacteria* (41.38%), *Actinobacteria* (20.08%) and *Acidobacteria* (13.74%) were the highest in proportion, compared with *Proteobacteria* (50.43%), *Actinobacteria* (29.59%) and *Bacteroidetes* (4.95%) in contaminated soil (Fig. 2A). Through *t* test analysis of gene abundance annotation, we found that the relative abundance of *Bacteroidetes* in the contaminated sample was significantly higher than that in the uncontaminated sample (*P* < 0.05; Fig. 2B). By contrast, the relative abundance of *Acidobacteria* was significantly lower than that in uncontaminated soil (*P* < 0.01; Fig. 2C). Oil pollution had significantly promoted the proliferation of *Bacteroidetes* and reduced the abundance of *Acidobacteria*, thus changed the distribution of soil microorganisms at the phylum level. We further analyzed the composition of soil microbial flora at genus level (Fig. 2D) and found that in uncontaminated soil, *Sphingomonas* took up 12.40% and *Nocardioides* 2.3%, while in contaminated soil, *Demequina* took up 4.02%, *Marinobacter* 3.82%, *Immundisolibacter* 3.95% and *Pseudoxanthomonas* 5.00%. Through *t* test analysis of gene abundance annotation, we found that the relative abundance of *Sphingomonas* was reduced in contaminated soil (*P* < 0.05; Fig. 2E), while those of *Pseudoxanthomonas*, *Pseudomonas* and *Mycobacterium* were increased (*P* < 0.01; Fig. 2F–G, Fig. S1). The results showed that petroleum pollution was able to inhibit the proliferation of *Sphingomonas* but promote those of *Pseudoxanthomonas*, *Pseudomonas* and *Mycobacterium*. This provides a reference basis for screening potential microorganisms for the bioremediation of contaminated soil.

### Analysis of functional genes annotation and metabolic pathways

The composition of microorganisms is often closely related to functions. This study further used KEGG database to annotate and classify the gene functions of the two soils and noticed significant differences in the overall functions of the two groups (PC1 52.46%, PC2 17.64%), which indicates that oil pollution changes not only the composition of microorganisms in the soil, but also their functions (Fig. S2A). A differential functional gene analysis of both groups was carried out. It was found that compared with uncontaminated soil, the contamination significantly reduced environmental information processing, organismal systems, metabolism, cellular processes and genetic information processing (Fig. S2B). More specifically, the abundance of genes in pathways of membrane transport, glycan biosynthesis and metabolism, xenobiotics biodegradation and metabolism, and environmental adaptation was decreased in contaminated soil. The results suggest that oil pollution may affect the communication between soil microbiome and the environment.

**Fig. 3:**
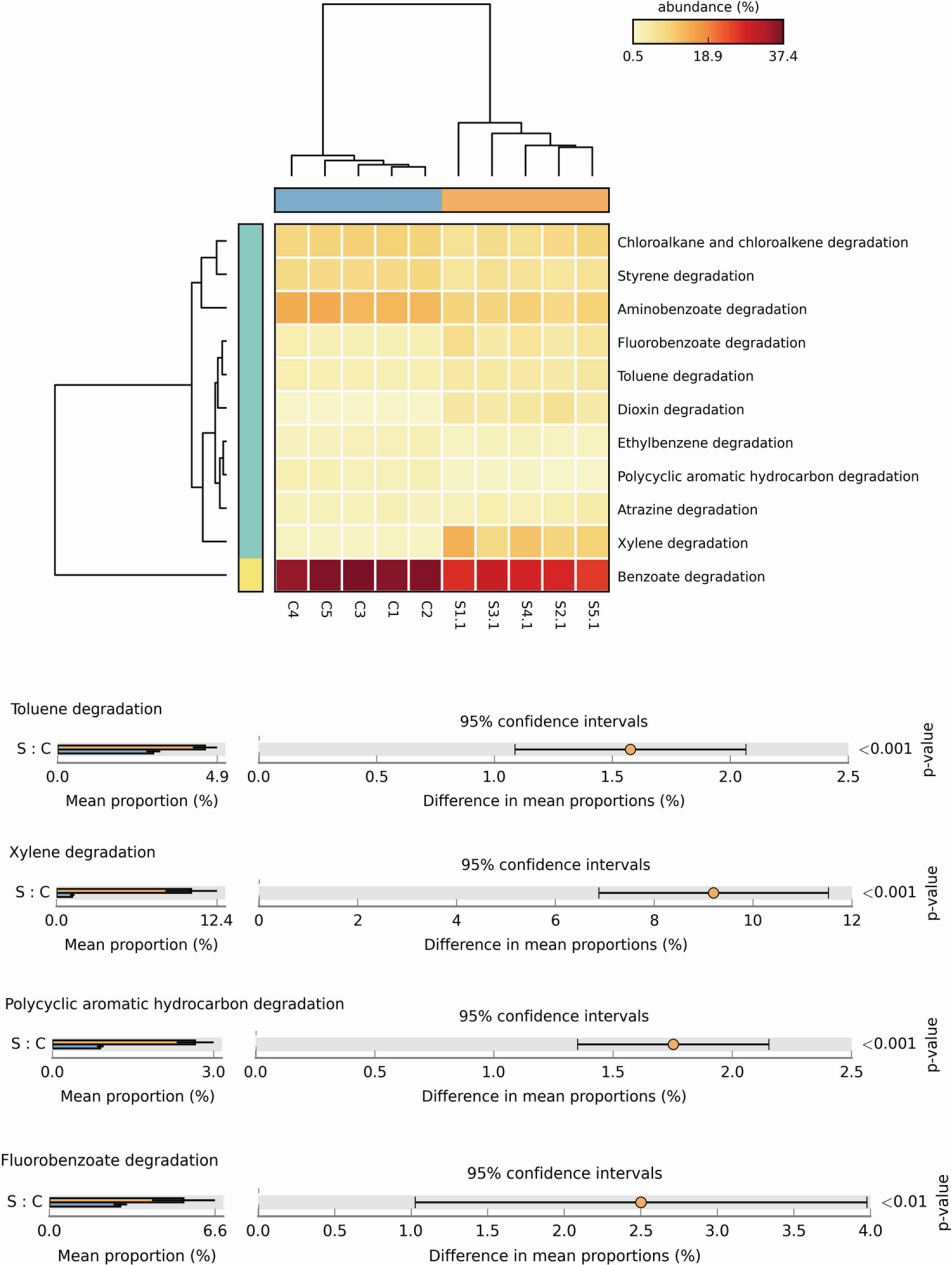
Differential pathways of xenobiotics biodegradation and metabolism. The heatmap (upper) showed significant differences in the number of genes in the related pathways. Significant difference enrichment pathways are screened based on Metastat method (lower half)

Microorganisms could also facilitate xenobiotic biodegradation and metabolism to protect soil from exogenous pollutants, such as oil and heavy metals [23]. In view of the fact that degradation pathways and regulating enzymes of foreign compounds by microorganisms in oil-contaminated soil are important in the study, we also focused on the difference analysis between the two groups on the pathway of xenobiotics biodegradation. It was found that the abundance of contaminated soil in degradations of toluene, xylene, polycyclic aromatic hydrocarbon and fluorobenzoate was significantly higher than that of uncontaminated soil (Fig. 3). These pathways were mainly related to the metabolism of aromatic hydrocarbons with benzene ring structure. Aromatic hydrocarbons are unsaturated hydrocarbon compounds, which are important components in petroleum. The chemical properties of their components are closely related to the toxic effect of petroleum pollution.

### Analysis of monooxygenase/dioxygenase code genes and resistance genes

**Fig. 4:**
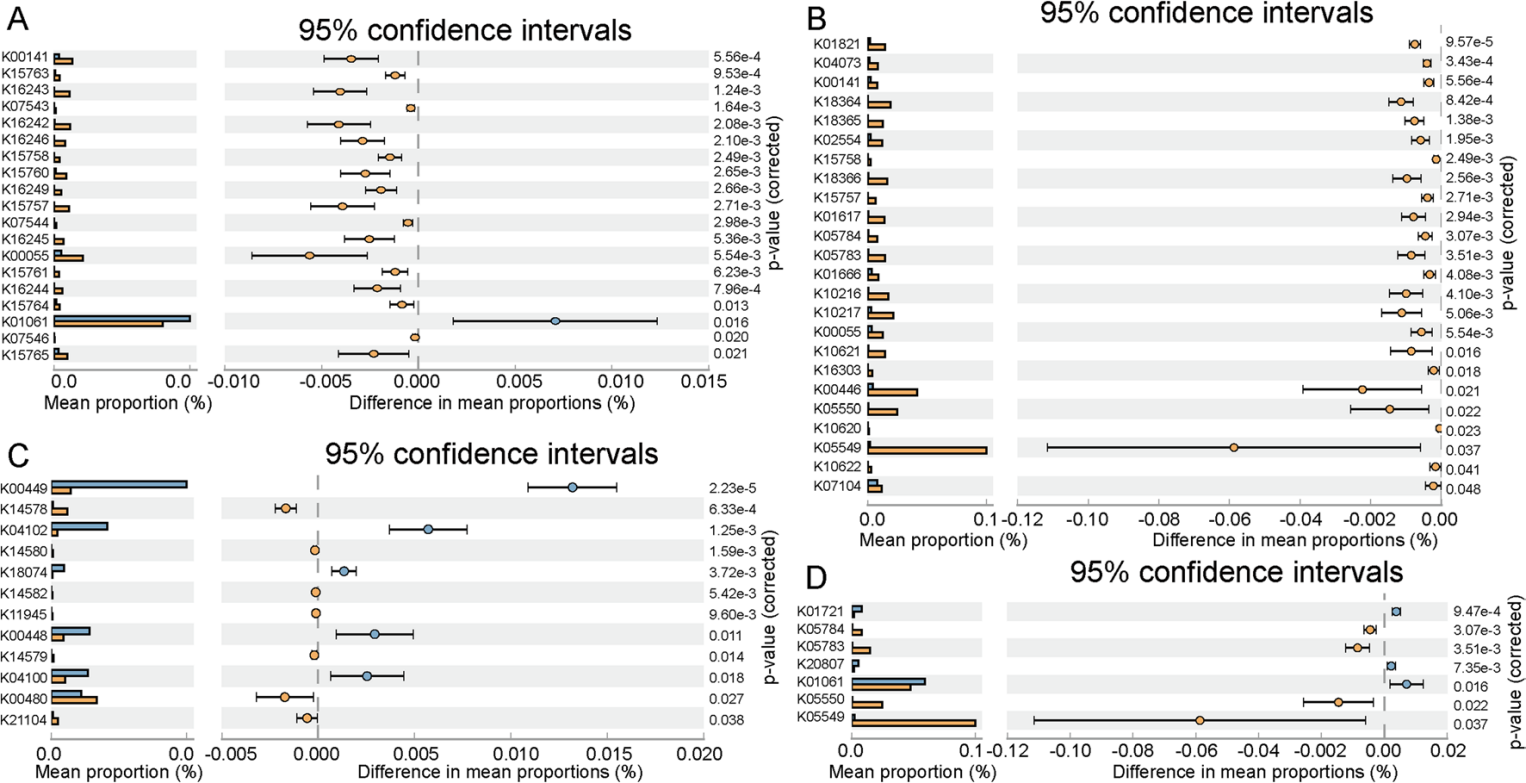
Differential KO analysis of petroleum-contaminated soil. **A–D** Represent the differential KO of toluene degradation, xylene degradation, PAH degradation and fluorobenzoate degradation in xenobiotics biodegradation and metropolis, respectively

Based on this, we further searched the above-mentioned differential metabolic pathways. It was found that 21, 24, 12 and 7 KOs (KEGG Orthology) were significantly different in differential metabolic pathways of degradations of toluene, xylene, polycyclic aromatic and fluorobenzoate. In the pathway of toluene degradation (Fig. 4A), the monooxygenase system significantly increased, including xylM, toluene methyl-monooxygenase, xylA, toluene methyl-monooxygenase electron transfer component, tmoA, tbuA1, touA, toluene monooxygenase system protein A, etc. Similarly, the gene abundance of dioxygenase and monooxygenase systems in xylene degradation pathway also increased (Fig. 4B) including benA xylX, benzoate/toluate 1,2-dioxygenase subunit alpha, benB-xylY, benzoate/toluate 1,2-dioxygenase subunit beta, etc. Dioxygenase significantly increased in the degradation pathway of PAHs (Fig. 4C), including nahAb, nagAb, ndoA, nbzAb, dntAb, nahAc, ndoB, nbzAc, dntAc, nahAd, ndoC, nbzAd, dntAd, phdF, nahB, doxE. In fluorobenzoate degradation (Fig. 4D), the abundance of the following genes increased. These are: benA xlX benzoate/toluate 1,2-dioxygenase subunit alpha, benB-xylY; benzoate/toluate 1,2-dioxygenase subunit beta, benD-xylL; dihydroxycyclohexadiene carboxylate dehydrogenase, benC-xylZ; benzoate/toluate 1,2-dioxygenase reductase component. Monooxygenase or dioxygenase is the key enzyme for aromatic hydrocarbon degradation. The specific KO found in this study provides a further theoretical reference for microbial degradation of oil pollution.

We further annotated and analyzed resistance genes through CARD database. Compared with uncontaminated soil, the abundance of resistance genes in the contaminated sample was higher and mainly concentrated in tlrB_conferring_tylosin, OXA-421, APH3-Ia, Pseudomonas_aeruginosa_soxR, aadA6, LRA-18, vanRE,vatF, PmrF, aadA5, mexQ, etc. (Fig. S3). The results indicated that oil-contaminated soil significantly increased the abundance of resistance genes in soil microorganisms.

### Composition of total metabolites in petroleum-contaminated soil

**Fig. 5:**
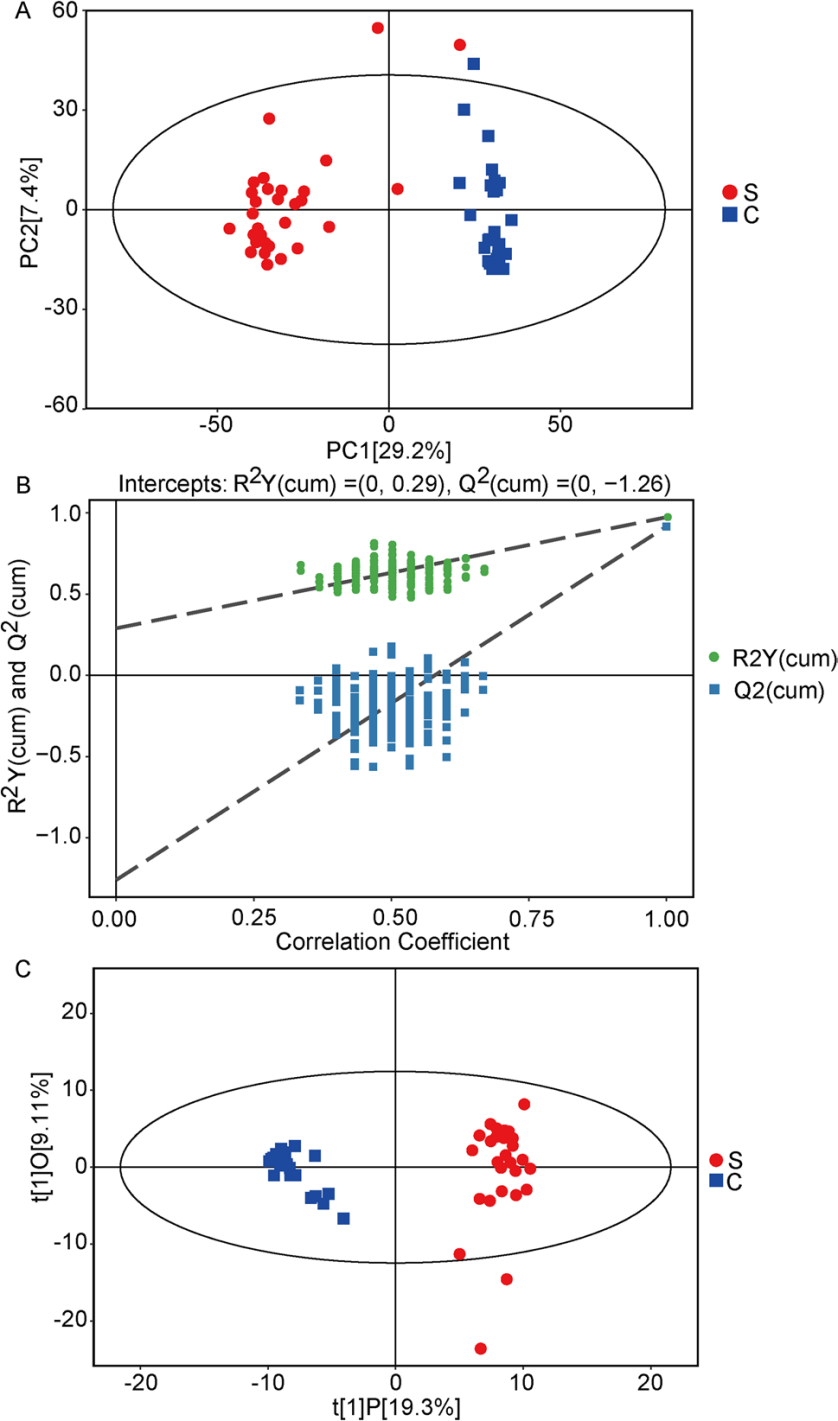
Petroleum-contaminated soil alters the distribution and composition of metabolites in soil. **A** The PCA score plots of the GC-TOFMS metabolites. **B** The PCA score chart of the GC-TOFMS metabolites obtained from the contaminated and uncontaminated soil. **C** Two hundred randomly arranged substitution tests were performed between contaminated and uncontaminated soil in the OPLS-DA model

Microorganisms are closely related to metabolites. Since we have found that petroleum contamination caused significant changes in microbial composition and speculated that metabolites would also change accordingly, we measured and analyzed non-targeted metabonomics data by GC-TOF–MS. We detected 378 peaks and 153 metabolites by relative standard deviation de-noising. Normalization of peak areas was performed. PCA showed significant differences in the overall distribution of metabolites between uncontaminated and contaminated soil (Fig. 5A), with 29.2% of variation being explained by the primary variable component PC1, and 7.4% by the primary variant component PC2. The results showed that petroleum pollution significantly changed the composition of soil metabolites. In order to further analyze the metabolite composition of petroleum-contaminated soil, we carried out OPLS-DA statistical method to analyze the results and obtained more reliable information about the correlation of the difference of metabolites between the two groups. The validity was evaluated by R2Y (the interpretability of the model to the classified variable y) and Q (the predictability of the model) obtained by cross validation. R2Y value was 0.29, and the corresponding Q2 value was − 1.26, indicating that the model is available and the data are stable and accurate (Fig. 5B). The two samples distribute clear, while the variations explained by PC1 and PC2 are 19.3% and 9.11%, respectively (Fig. 5C).

### Differential metabolites in contaminated soil

**Fig. 6:**
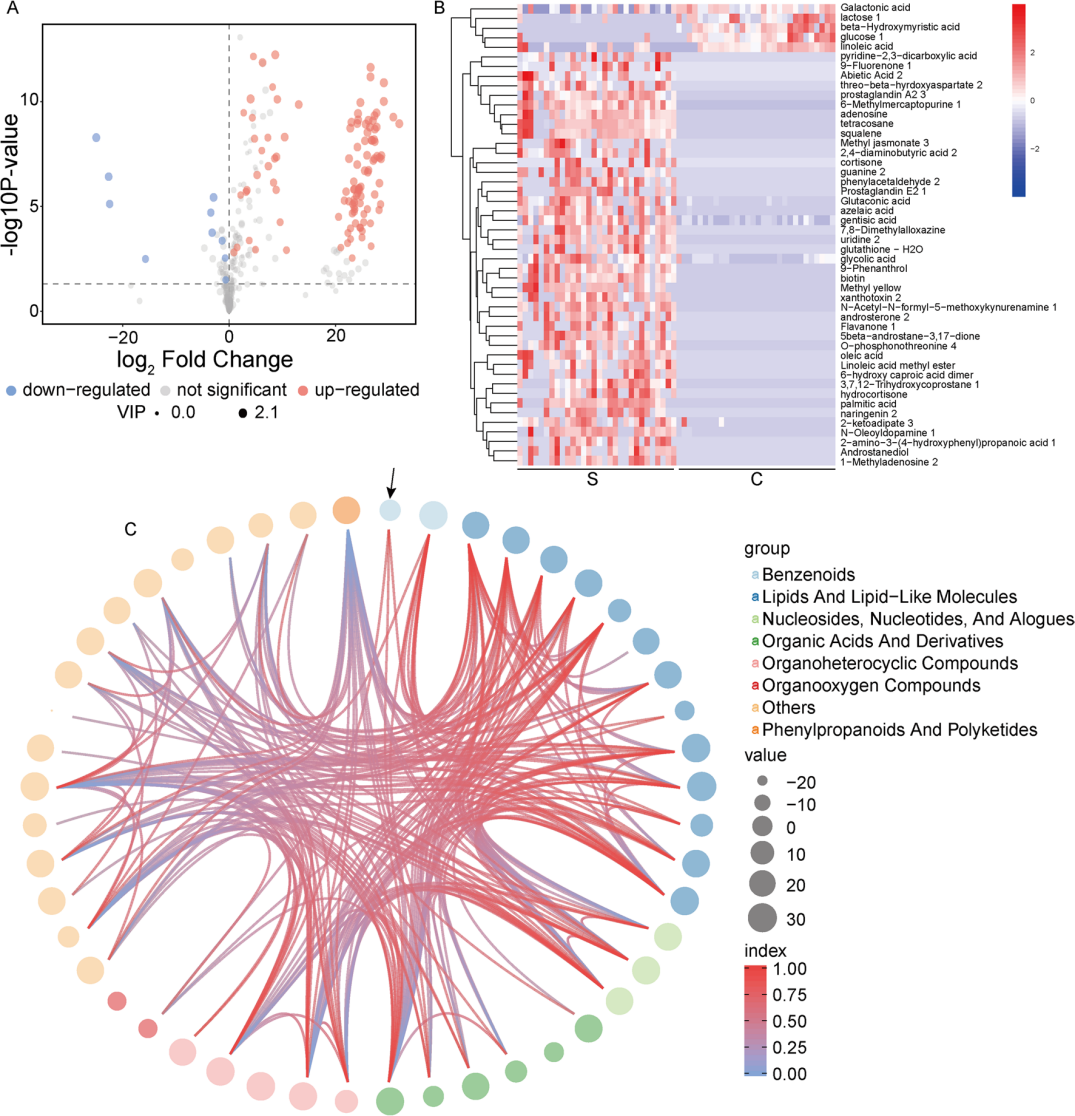
Specific metabolites and cluster analysis of petroleum-contaminated soil. **A** Volcano diagram of differential metabolites between two groups. **B** Metabolite profiling of contaminated and uncontaminated soil. The color blocks in different positions represent the relative expression of metabolites in corresponding positions. Red indicates high expression of metabolites, and blue indicates low expression of metabolites. **C** Chord plot for the analysis of contaminated and uncontaminated soil. Starting from the arrow-pointed circle in a clockwise direction, the metabolites are: benzenoids (gentisic acid and phenylacetaldehyde 2); lipids and lipid-like molecules (3,7,12-trihydroxycoprostane 1, 5beta-androstane-3,17-dione, androstanediol, androsterone 2, azelaic acid, cortisone, hydrocortisone, linoleic acid, oleic acid, palmitic acid, prostaglandin a2 3, prostaglandin e2 1 and squalene); nucleosides, nucleotides and alogues (1-methyladenosine 2, adenosine and uridine 2); organic acids and derivatives (6-hydroxy caproic acid dimer, galactonic acid, glutaconic acid, glutathione-h2o, glycolic acid and o-phosphonothreonine 4); oganoheterocyclic compounds (6-methylmercaptopurine 1, biotin, guanine 2, methyl yellow and pyridine-2,3-dicarboxylic acid); organooxygen compounds (glucose 1 and lactose 1); others (2-amino-3-(4-hydroxyphenyl) propanoic acid 1, 2-ketoadipate 3, 2,4-diaminobutyric acid 2, 7,8-dimethylalloxazine, 9-fluorenone 1, 9-phenanthrol, abietic acid 2, beta-hydroxymyristic acid, flavanone 1, linoleic acid methyl ester, methyl jasmonate 3, n-acetyl-n-formyl-5-methoxykynurenamine 1, n-oleoyldopamine 1, naringenin 2, tetracosane and threo-beta-hyrdoxyaspartate 2); phenylpropanoids and polyketides (xanthotoxin 2)

The results of the screened differential metabolites showed that compared with uncontaminated soil, ten metabolites in contaminated soil were significantly down-regulated, including glucose, linoleic acid, lactose, galactic acid and beta hydroxycholic acid, while 143 metabolites significantly increased, such as palmitic acid, tetracosane, 2-ketoadipate, glycolic acid, azelaic acid and methyl jasmonate (Fig. 6A).

The different metabolites obtained from the above analysis often demonstrate similar results and functions in biology and are regulated by the same metabolic pathway. Therefore, this study further performed a cluster analysis of the differential metabolites (Fig. 6B). The main metabolites in contaminated soil were pyridine-2, 3-dicarboxylic acid, 9-fluorenone, abietic acid, threo-beta-hydroxyaspartate, prostaglandin A2, 6-methylmercaptopurine, adenosine, azelaic acid, gentisic acid, 7,8-dimethylalloxazine, uridine, glutathione, glycolic acid, 9-phenanthrol, biotin, etc., which fall into the following broader categories of metabolism: benzenoids, palmitic acid, oleic acid, nucleotides, organic acids and their derivatives, organoheterocyclic compounds, organooxygen compounds, phenylpropanoids and polyketides (Fig. 6C). These results indicated that petroleum contamination can promote the metabolism of benzenoids, lipids and lipid-like molecules, as well as organoheterocyclic compounds and phenylpropanoids. In addition, gentian acid was one of the end products of microbial degradation of PAHs. And 9-fluorenone was formed by oxidation and dehydrogenation of fluorene at C9 position, which indicates a possible degradation pathway of various polycyclic aromatic hydrocarbon pollutants by soil microorganisms. This also verifies that microorganisms in oil-contaminated soil have the ability to degrade PAHs.

## Discussion

**Fig. 7:**
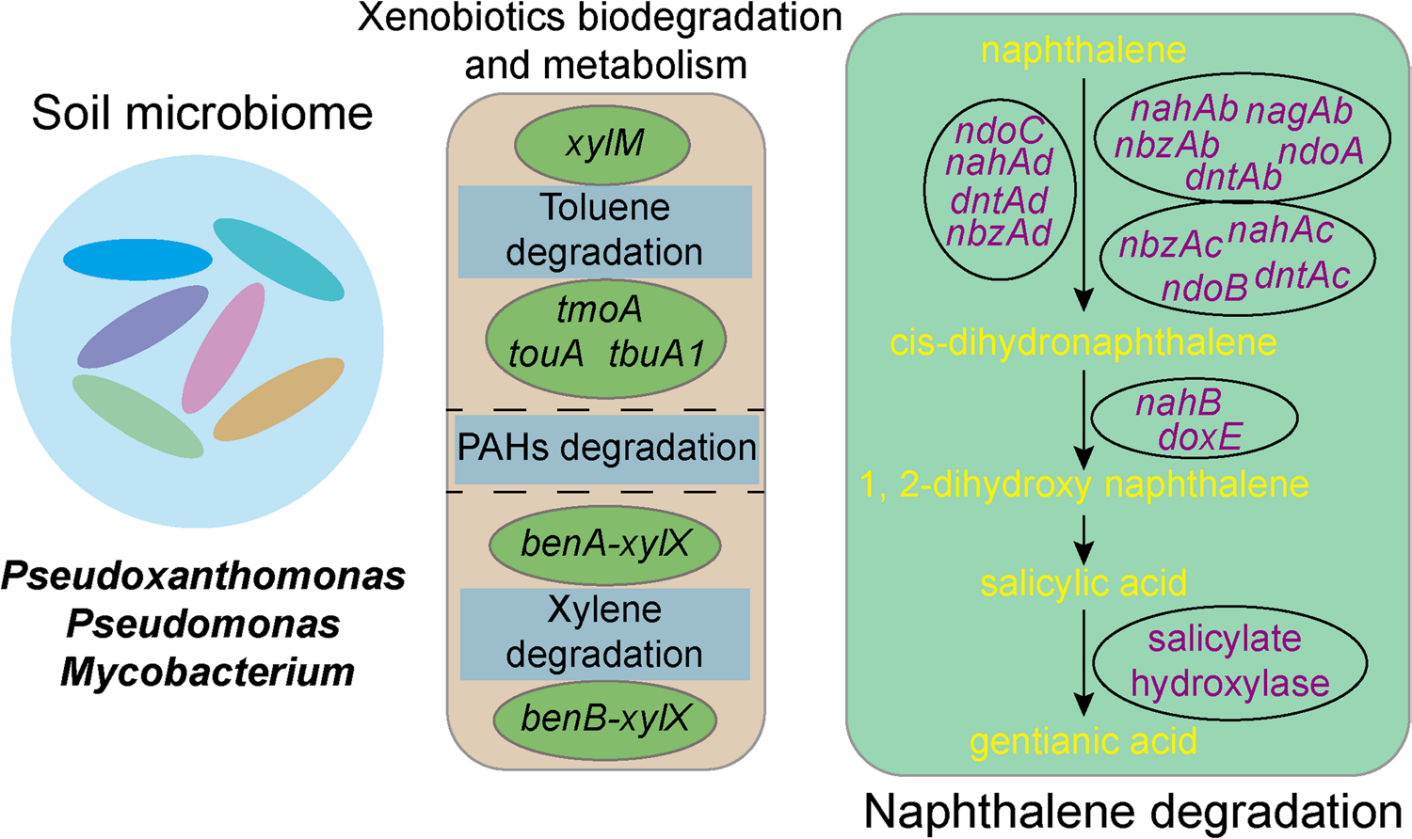
Petroleum-contaminated changes the flora, function and metabolism of soil

In this study, metagenomic and metabonomic were used to detect the microbes and metabolites in oil-contaminated soil, and the changes of functional pathways were analyzed. It was found that the composition and metabolite distribution of microbes changed significantly in oil-contaminated soil, in which the relative abundances of *Pseudoxanthomonas*, *Pseudomonas*, *Mycobacterium*, etc. were increased. The degradation of the following compounds increased, which are toluene, xylene, polycyclic aromatic hydrocarbon and fluorobenzoate. The metabolism and regulation of key monooxygenase and dioxygenase systems to promote ring opening and degradation of aromatic hydrocarbons in petroleum also increased, as well as metabolite contents of PAHs such as 2-ketoadipate, 9-fluoronone, generic acid, 9-phenanthrol (Fig. 7). The pollution of petroleum hydrocarbons to soil can increase the number and species of microorganisms that are able to degrade petroleum hydrocarbons itself. Bacteria and fungi are the main microorganisms that can degrade petroleum hydrocarbons in soil [24]. Bhuyan et al. [25] isolated two strains of *Pseudomonas aeruginosa*, which have high efficiency of petroleum hydrocarbons degradation and plant growth promotion ability, and could be used for subsequent bioremediation of crude oil-contaminated soil. Kumari et al. [26] investigated the ability of *Pseudomonas aeruginosa* to degrade multiple polycyclic aromatic hydrocarbons in crude oil and found that *Pseudomonas aeruginosa* had a high biodegradation rate for specific PAHs in petroleum, such as 67.1% for phenanthrene and 61.0% for benzo(b)fluoranthene. Banerjee et al. [27] isolated a new *Pseudomonas* strain, which can effectively degrade benzene, toluene, meta- and para- xylene effectively under both aerobic and microaerobic conditions, from groundwater contaminated by residual petroleum hydrocarbons. *Pseudoxanthomonas* is also considered to have potential applications in bioreactive strategies. It was found that *Pseudxanthomonas*, with the best degrading ability among the 25 isolated phenanthrene-degrading strains, could use other exogenous compounds as the only carbon source to reduce phenanthrene [28]. *Pseudoxanthomonas* is highly effective in degrading pyrene in soil [29]. *Mycobacterium* is used in repairing soil contaminated by PAEs and shows a high degradation efficiency in water systems containing phenanthrene, pyrene, benzo(a)pyrene and benzo(b)fluoranthrene [30]. In addition, *Demequina*, *Marinobacter* and *Immundisolibacter*, which are highly abundant bacteria genera in petroleum-contaminated soil, are important in degrading petroleum hydrocarbons, polycyclic aromatic hydrocarbons and other harmful contaminants [14, 31, 32]. Recent studies have shown that *Immundisolibacter* is a key microorganism in benz(a)anthracene degradation [31]. In this study, the changes of microbial community in oil-contaminated soil were explored, and the detected biomarkers may provide new insights for identifying potential microorganism that are capable of biodegrading petroleum contaminants.

These results indicate that these strains have great potential for bioremediation of contaminated soil from gas stations. In this study, it was found that petroleum contamination significantly promoted the proliferation of *Pseudoxanthomonas*, *Pseudomonas*, *Mycobacterium*, *Immundisolibacter*, etc., which have been widely studied as strains capable of degrading aromatic hydrocarbons in petroleum [33, 34]. The mechanism of microbial degradation of petroleum pollutants is to regulate relevant enzymes to decompose aromatic hydrocarbons and absorb carbon source materials necessary for life activities through their own metabolic activities, and the energy generated in the metabolic process can be used to carry out various life activities. Aromatic hydrocarbon dioxygenases are important because they can initiate microbial aerobic degradation of these compounds [30, 35]. Dioxygenase is the speed limiting factor of aromatic hydrocarbon oxidation ring opening, so dioxygenase gene polymorphism and its content are often used as detection standard for the self-repair ability of contaminated soil [36]. The degradation of aromatic hydrocarbons by microorganisms can be divided into two types: anaerobic and aerobic. Aerobic oxidation under aerobic conditions is the main way of aromatic hydrocarbon degradation. All aromatic hydrocarbon substances contain benzene ring structure. So their degradation processes share a common character, that is, monooxygenase or dioxygenase catalyzes the oxygenation, then the benzene ring structure opens. The product is then involved in the Trifusic acid cycle, and degradation completes [37]. When aromatic hydrocarbon pollutants enter the environment, microorganisms are stimulated to produce and secrete monooxygenase or dioxygenase [38]. The dioxygenase can directly add oxygen molecules to the PAHs to form peroxides. This process is also known as aryl double functionalization [39]. Through this process, the opening of the aromatic ring becomes very easy, and then degradation is completed. At present, this method has gradually become the most potential means to repair aromatic hydrocarbons in soil environment. In this study, it was found that microorganisms in the oil pollution group could regulate a variety of key monooxygenase and dioxygenase systems to promote ring opening and degradation of aromatic hydrocarbons in oil. For example, in the xylene degradation pathway, catE, benA xylX, etc. were significantly up-regulated. In the polycyclic aromatic degradation pathway, nahAb, nahAc, nagAb, etc. were significantly up-regulated, indicating that the degradation of PAHs mainly aimed at naphthalene and other compounds. This part of the genes was consistent with the reported genes that can degrade naphthalene. In addition, in this study, phdF and other factors related to phenanthrene degradation were not significantly up-regulated, and there was no significant difference between the two groups. Therefore, microbes collected from petroleum-contaminated soil here mainly up-regulated the degradation of naphthalene.

The degradation of naphthalene starts from the formation of cis-dihydronaphthalene by attacking the aromatic ring with dioxygenase, then comes 1, 2-dihydroxy naphthalene through the action of dehydrogenase. Through a series of catalytic reactions, salicylic acid forms, which is then decomposed to form gentianic acid, after which ring-opening degradation completes [40]. This study further determined the metabolites of oil-contaminated soil samples through metabolome technology and found no catechol production. However, the content of gentian acid was significantly higher than that of the control group, indicating that in this soil sample, microorganisms mainly degraded naphthalene through the hydroxylated gentian acid pathway. At the same time, compared with differential KOs in the above macro genome, it was found that nahAb, nagAb, ndoA, nbzAb, dntAb, nahAc, ndoB, nbzAc, dntAc, nahAd, ndoC, as well as the abundance of naphthalene dioxygenases such as nbzAd and dntAd, were significantly up-regulated, while the abundance of nahB, doxE dehydrogenase and salicylate hydroxylase increased, which further verified the complete pathway of naphthalene degradation mentioned above. Leneva et al. [41] used HPLC, TLC and mass spectrometry to detect the naphthalene degradation intermediates of *Pseudomonas fluorescens* 26 K strain. Identified metabolites include naphthalene cis-1,2-dihydrodiol, salicylaldehyde, salicylate, catechol, 2-hydroxymuconic semialdehyde and 1-naphthol. Catechol 2,3-dioxygenase and cis-1,2-dihydrodiol dehydrogenase with native molecular mass 160 kDa were purified from crude extract of the strain and characterized. On this basis, they proposed the pathway of naphthalene degradation by *Pseudomonas* sp. Benedek et al. [42] found regardless of oxygenation condition, *Pseudomonas* spp. became the most dominant in the naphthalene-amended selective enrichment cultures.

Resistance genes are ubiquitous in soil microorganisms. The abuse of antibiotics leads to irreversible changes to the microbial community of human body and environment, and causes risks to the ecological environment [43]. This study also found that oil pollution promoted the abundance of resistance genes in soil. Das et al. [44] found that the functional abundance of genes related to antibiotic resistance in petroleum-contaminated samples was more than twice, and the abundance of genes related to fluoroquinolones in contaminated samples was as high as 10 times through metagenomic analysis. These high abundance antibiotic resistance genes indicated that petroleum hydrocarbon-contaminated sites could be considered a pool of antibiotic resistance genes. Zhang et al. [45] found that exhaust particulates of gasoline and diesel (97 octane gasoline, 93 octane gasoline, light diesel and marine heavy diesel) could accelerate the transfer of antibiotic mediated resistance genes. This discovery provides new evidence and mechanism insights for the risk of antimicrobial resistance of gasoline and diesel exhaust particulates, and also shows that the transfer and increase of drug resistance genes is one of the hazards of oil pollution. From this study, we get that there is an important relationship between the existence of petroleum hydrocarbons and the development of antibiotic resistance in the contaminated microbial community.

## Conclusion

In conclusion, based on metagenomics technology, this study found that *Pseudoxanthomonas*, *Pseudomonas*, *Mycobacterium*, *Immundisolibacter*, etc. were the dominant degradation bacteria in oil-contaminated soil. Through functional analysis, we clarified the relevant mechanism of microbial degradation of aromatic compounds, further analyzed the difference of products through metabolomics technology and found the relevant degradation characteristics of microorganisms on PAHs, toluene and other petroleum pollutants. The microorganisms also regulate the production of related metabolites, such as gentian acid and 9-fluorenone. This study provided theoretical basis and treatment measures for the bioremediation of PAHs. It will also generate fresh insight into the screening of new oil degrading bacteria.

## Supplementary Information

Below is the link to the electronic supplementary material.

Supplementary file1 (DOCX 295 KB)

## Data Availability

All data analyzed during this study are included in this published article. The raw data of this study are available from the author Yoanquan Li upon reasonable request.
