# Supplementary material for: Metagenomics-metabolomics analysis of microbial function and metabolism in petroleum-contaminated soil
Source: Braz J Microbiol. 2023 May 10;54(2):935–47. doi: 10.1007/s42770-023-01000-7 (PMC10234942; doi:10.1007/s42770-023-01000-7)
Supplement: Supplementary file 1 — Supplementary file1 (DOCX 295 KB) [file 42770_2023_1000_MOESM1_ESM.docx]

**Supplementary materials**


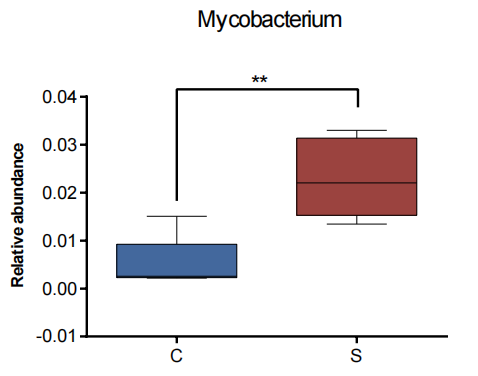


**Fig. S1 The relative abundances of *Mycobacterium***


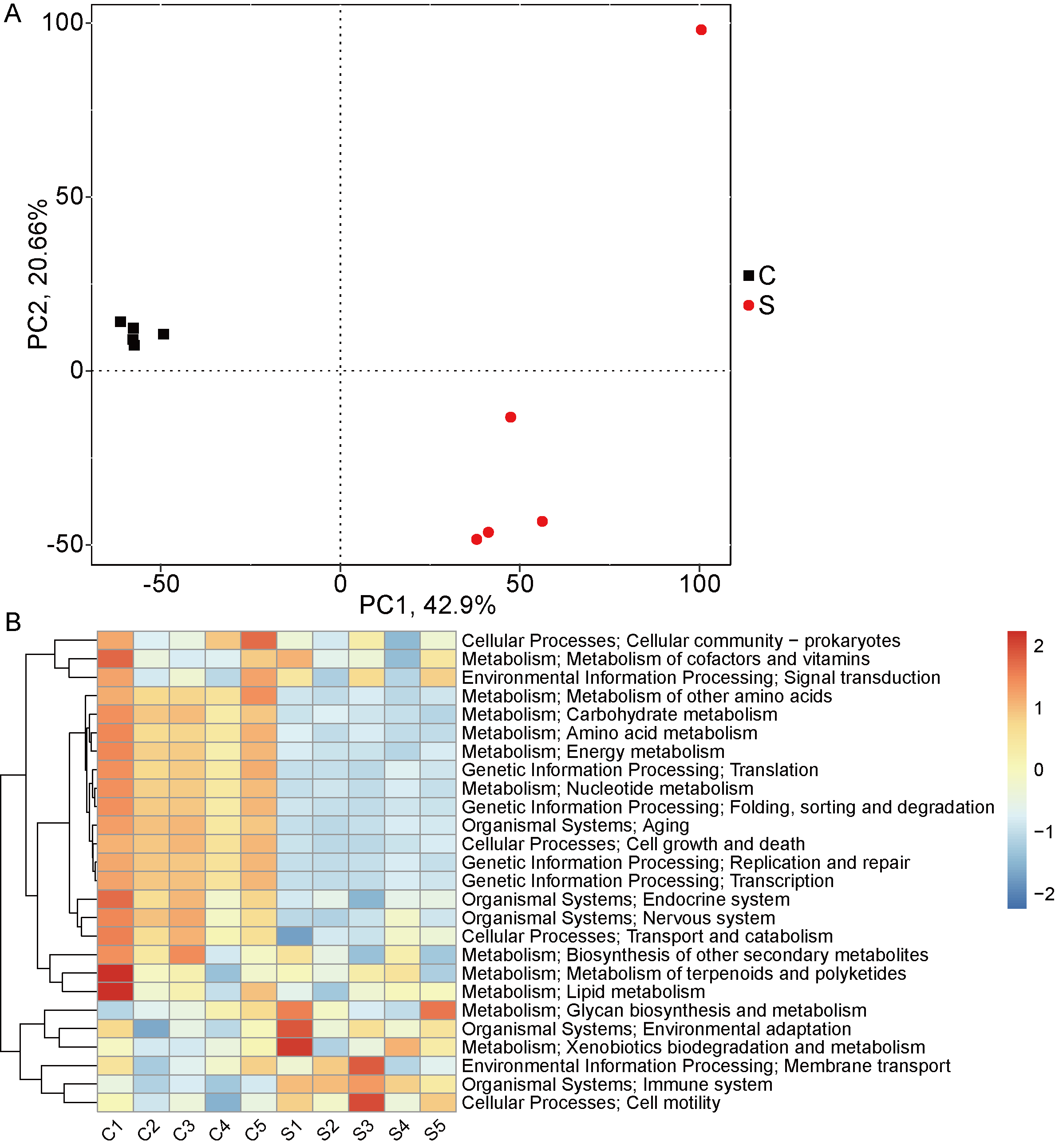


**Fig. S2 Functional gene annotation and metabolic pathway analysis**

**A** PCA analysis results displayed based on KEGG function abundance; **B** Clustering analysis based on level 2 of KEGG database.


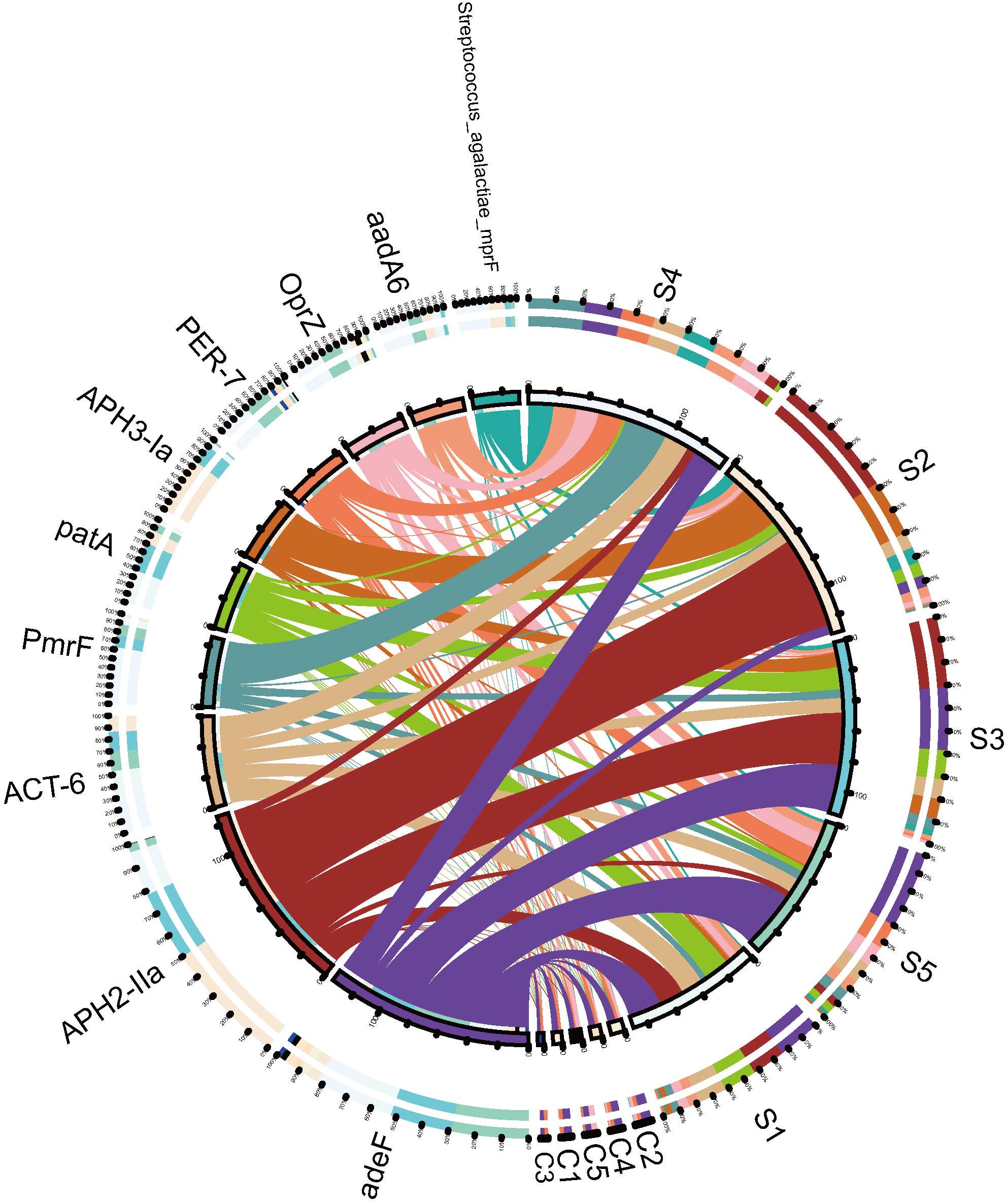


**Fig. S3** Overview of the distribution ratio of resistance gene types in groups S and C. Different colors in the inner circle indicate different samples and resistance genes. The left side is the sum of the relative abundance of resistance genes of all samples, while the right side is the relative abundance of resistance genes in one sample.
